# 印戒细胞样浆细胞大量增生的多发性骨髓瘤1例

**DOI:** 10.3760/cma.j.cn121090-20250418-00190

**Published:** 2025-12

**Authors:** 志瑶 白, 艳玲 晋, 雪 吴, 帆 张, 鹤倩 刘, 慕然 杨, 萍 吉

**Affiliations:** 1 昆明医科大学附属曲靖医院医学检验中心，曲靖 655000 Center for Laboratory Medicine, Qujing Hospital of Kunming Medical University, Qujing 655000, China; 2 昆明医科大学附属曲靖医院血液科，曲靖 655000 Department of Hematology, Qujing Hospital of Kunming Medical University, Qujing 655000, China; 3 同济大学附属同济医院检验科，上海 200065 Department of Laboratory Medicine, Shanghai Tongji Hospital, School of Medicine, Tongji University, Shanghai 200065, China

患者，男，68岁，因“腰背疼痛4个月，加重伴咳嗽3 d”入院。体格检查无异常，CT示脑颅骨、双侧肋骨及腰椎多发溶骨性破坏伴椎体压缩性骨折。实验室检查：血常规正常；钙2.24 mmol/L，肌酐68 µmol/L，血尿素氮11.7 mmol/L；血清总蛋白93.1 g/L，白蛋白22.2 g/L，球蛋白70.9 g/L，IgG 53.7 g/L，IgA<0.26 g/L，IgM<0.19 g/L，β_2_微球蛋白4.1 mg/L。虽未满足多发性骨髓瘤（MM）的典型表现（高钙血症、肾功能不全、贫血或骨病变等终末器官损害），但球蛋白显著升高，提示可能存在单克隆免疫球蛋白异常。血清蛋白电泳示M蛋白占50.9％，免疫固定电泳示IgG κ型，尿本周蛋白阳性；血清游离轻链κ/λ比值149.19，尿液游离轻链κ/λ比值为23.57。骨髓穿刺：浆细胞约占53％，大小不一，多数胞质内可见巨大圆球形透明空泡，部分细胞质内见1～3个大小不等空泡，细胞核被挤压至胞质的一侧，形似印戒细胞，偶见双核印戒样细胞（图1）。流式细胞术免疫分型：单克隆浆细胞占25.63％，CD38^+^、CD138^+^、CD19^-^、CD20^-^、CD28^-^、CD56^-^、CD81^-^、CD200^+^，细胞内Ig轻链κ限制性表达。骨髓活检：浆细胞呈片状分布，约占65％，可见双核及多核浆细胞，免疫组化CD38（65％强+，图2A），CD138（65％片状+，图2B），κ（部分弱+，图2C），λ（偶见+，图2D），CD117（偶见+），CD20（少见+），CD56（偶见+），BCMA（50％，中等+），计数200个浆细胞，其中κ阳性细胞199个，λ阳性细胞1个，κ/λ＝199∶1，诊断意见：符合浆细胞骨髓瘤。综合诊断为IgG κ型MM，Durie-Salmon分期Ⅲ期A组。明确诊断后，患者接受VAD方案（长春新碱+阿霉素+地塞米松）化疗约4个月后获得部分缓解，随后调整为KPD方案（卡非佐米+泊马度胺+地塞米松）化疗，目前已接受KPD方案治疗约1个月，病情稳定，身体状况良好。

**图1:**
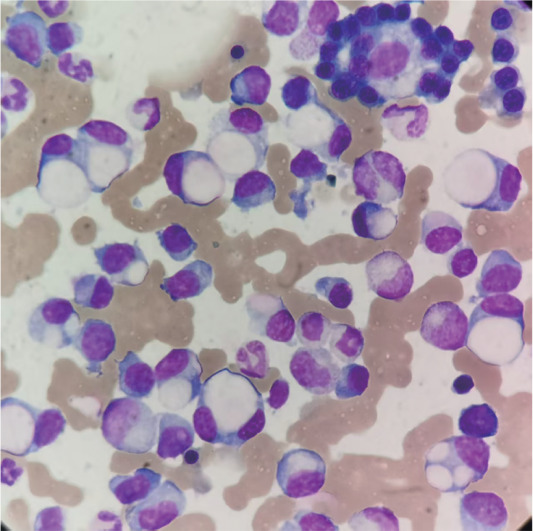
患者骨髓细胞形态学示大量印戒细胞样浆细胞（Wright法，×1 000）

**图2:**
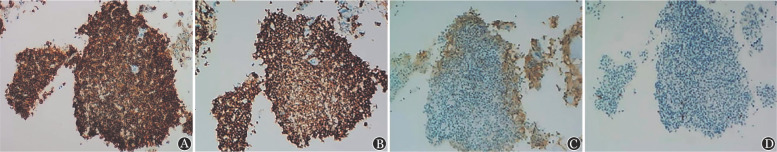
患者骨髓活检免疫组织化学染色结果（EnVision法，×100） **A** CD38肿瘤细胞弥漫阳性；**B** CD138肿瘤细胞弥漫阳性；**C** 部分肿瘤细胞κ阳性；**D** 部分肿瘤细胞λ偶见阳性

讨论：骨髓中异常浆细胞是MM重要的诊断依据。临床上较为常见的异常浆细胞形态学特点为：①浆细胞样骨髓瘤细胞；②网状细胞样骨髓瘤细胞；③不规则形骨髓瘤细胞，包括含有不同包涵体的浆细胞，如Auer样杆状小体、Russell小体、Mott细胞等。本例患者18％的浆细胞呈现印戒样变（胞质内巨大透明包涵体挤压核偏位），这一现象在MM中极为罕见，全球仅少数病例报道。印戒样细胞常见于产生黏蛋白的腺癌（如胃癌转移）或B细胞淋巴瘤，其形态学特征易导致其被误诊为转移性肿瘤。目前认为，浆细胞印戒样变的形成可能与免疫球蛋白组装缺陷有关，异常蓄积的免疫球蛋白形成胞质包涵体，迫使细胞核移位。鉴别诊断需结合免疫表型及M蛋白检测，以排除其他转移型印戒肿瘤细胞。

